# Electrical resistivity survey data for potential aquifer in Banggi Island, Sabah, Malaysia

**DOI:** 10.1016/j.dib.2020.106194

**Published:** 2020-08-18

**Authors:** Muhd Nur Ismail Abdul Rahman, Hafeez Jeofry, Mohd Sazaly Basarian

**Affiliations:** a Faculty of Science and Marine Environment, Universiti Malaysia Terengganu, 21030 Kuala Nerus, Terengganu Malaysia; b Institute of Oceanography and Environment, Universiti Malaysia Terengganu, 21030 Kuala Nerus, Terengganu, Malaysia; c Azair Sdn.Bhd. Lot No.2 Pusat Perindustrian Kelombong Jaya, Lorong Kacang Tanah 1, Mile 5.5, Jalan Kolombong, Kota KInabalu, Sabah Malaysia

**Keywords:** Aquifer, Banggi Island, Pole-dipole array, Bongaya formation, Chert-spilite formations, 2D resistivity model

## Abstract

The survey data on potential aquifer was collected at two sites located in Banggi Island (i.e. Laksian Primary School [LPS] and Padang Primary School [PPS]), Malaysia on 25 and 26 April 2013. Both locations are geologically surrounded by various types of lithologies, namely, sandstone, mudstone, siltstone, shale, chert, conglomerate, lignite, tuff, limestone, terrace sand, gravel and coral. The resistivity data consisted of six-line pole-dipole short arrays and were recorded in-situ using SAS 4000 ABEM Lund Imaging System, together with a relay switching unit (Electrode Selector ES 464), six multiconductor cables, steel rod electrodes and jumpers. The data, namely electrode spacing, depth of investigation, subsurface resistivity, type of material and horizontal data coverage were used to assess the characteristics of the potential aquifer. The recorded data were then processed using RES2DINV software to obtain 2-D inversion model of the subsurface. The data were also equipped with six models of inverse resistivity section for both areas. The data obtained can be used by the government and stakeholders for groundwater exploration and extraction in order to provide water supplies for local communities, especially since access to these resources from the surrounding water treatment plants on the island is limited.

**Specifications Table**

**Table:** 

**Subject**	Earth and Planetary Sciences
**Specific subject area**	Geophysics, Groundwater
**Type of data**	Figure, Table, Photo, DAT and INV files
**How data were acquired**	Data were obtained from Azair Sdn. Bhd (ASB), a private enterprise in water treatment and environmental concerns. The electrical resistivity survey using SAS 4000 ABEM Lund Imaging System
**Data format**	Raw, Processed
**Parameters for data collection**	Electrode spacing, depth of investigation, subsurface resistivity, type of material, horizontal data coverage.
**Description of data collection**	Raw data were obtained from the ASB, which contributed towards geophysical datasets of paper. The electrical resistivity survey was carried out using pole-dipole array method to obtain field data. Two locations were evaluated by establishing three line transects. A transect line was stretched up to 400 metres laterally and 220 metres vertically at LPS. For PPS, the transect line was stretched up to 400 metres vertically and 200 metres laterally. Every transect line employed different electrode, cable jumper and lund cables, i.e. every 400-metre stretch utilised 61 electrodes, 64 cable jumper and 4 rolls lund king cables. The 220-metre and 200-metre stretch utilised 41 electrodes with 42 cable jumpers and 2 rolls lund imaging cables. All the electrical resistivity data were typically successful acquisition using SAS 400 ABEM Lund Imaging System. The field data were subsequently inverted using RES2DINV software to acquire a 2-D inversion model of the subsurface.
**Data source location**	Geological Map of Sabah [1]. Department of Mineral and Geoscience Malaysia, Sabah Branch.
	Laksian Primary School, Banggi, Kudat, Sabah, Malaysia (N07°10′14.00″ E117°08′27.07″)
	Padang Primary School, Banggi, Kudat, Sabah, Malaysia
	(N07°15′34.20″ E117°04′14.30″)
**Data accessibility**	Public repository and with this article
	Repository: Mendeley data
	http://dx.doi.org/10.17632/x3mrhtxnby.1
	http://dx.doi.org/10.17632/x3mrhtxnby.2
	**DOI:**10.17632/x3mrhtxnby.1 and 10.17632/x3mrhtxnby.2

## 1 Value of the data

• The electrical resistivity data can be utilised for subsurface characterisation, assess lithologic layers, typically determine the specific depth and possible extent of potential aquifer bearing formations.
  Government and stakeholders may use the data to pinpoint the exact locations for potential aquifer sites on Banggi Island, and subsequently to propose a tubewell for the groundwater extraction.
• These data can be used to correlate with the logging profile data from other areas within the same island to reconstruct a complete groundwater model, in order to advance comprehension on groundwater research on Banggi Island.
• The electrical resistivity data can be used as baseline data for groundwater exploration and extraction for water supply resources to local communities on the tropical island, since access to treated water from treatment plants is limited.

## 2 Data description

Table 1 shows recorded data from both areas, LPS and PPS, respectively. The data, which consisted of electrode spacing, depth of investigation, subsurface resistivity, type of material and horizontal data coverage - were basically derived from field surveys, and subsequently processed via RES2DINV software. More recorded data of resistivity and detail electrode employed in this survey have stored in **public repository (Mendeley)**. The data attached files in public repository consist of electrical resistivity data sets used for subsurface evaluation. The raw data sets are presented in “.DAT’ format (DAT files) and the processed datasets are presented in ‘. INV’ format (INV files).

**Table 1:** Summary of recorded data of three survey lines in LPS and PPS areas.

LPS	Survey Line	Electrode Spacing, m	Depth of investigation, m	Resistivity of material, Ω-m	Vertical, m	Horizontal, m	Material
	1	5	150	1–100	–	400	Clay, silt
	2	5	150	1–100	–	400	Clay, clay, weathered rock
	3	5	80	1–100	220	–	Silt
PPS	Survey Line	Electrode Spacing, m	Depth of investigation, m	Resistivity of material, Ω-m	Vertical, m	Horizontal, m	Material
	1	5	80	1–100	–	200	Clay, silt, minor sandstone, coral
	2	5	80	1–100	–	200	Clay, silt, coral, weathered rock
	3	5	150	8–4000	400	–	Sandstone

## 3 Experimental design, materials, and methods

### 3.1 Study area

**Fig. 1:**
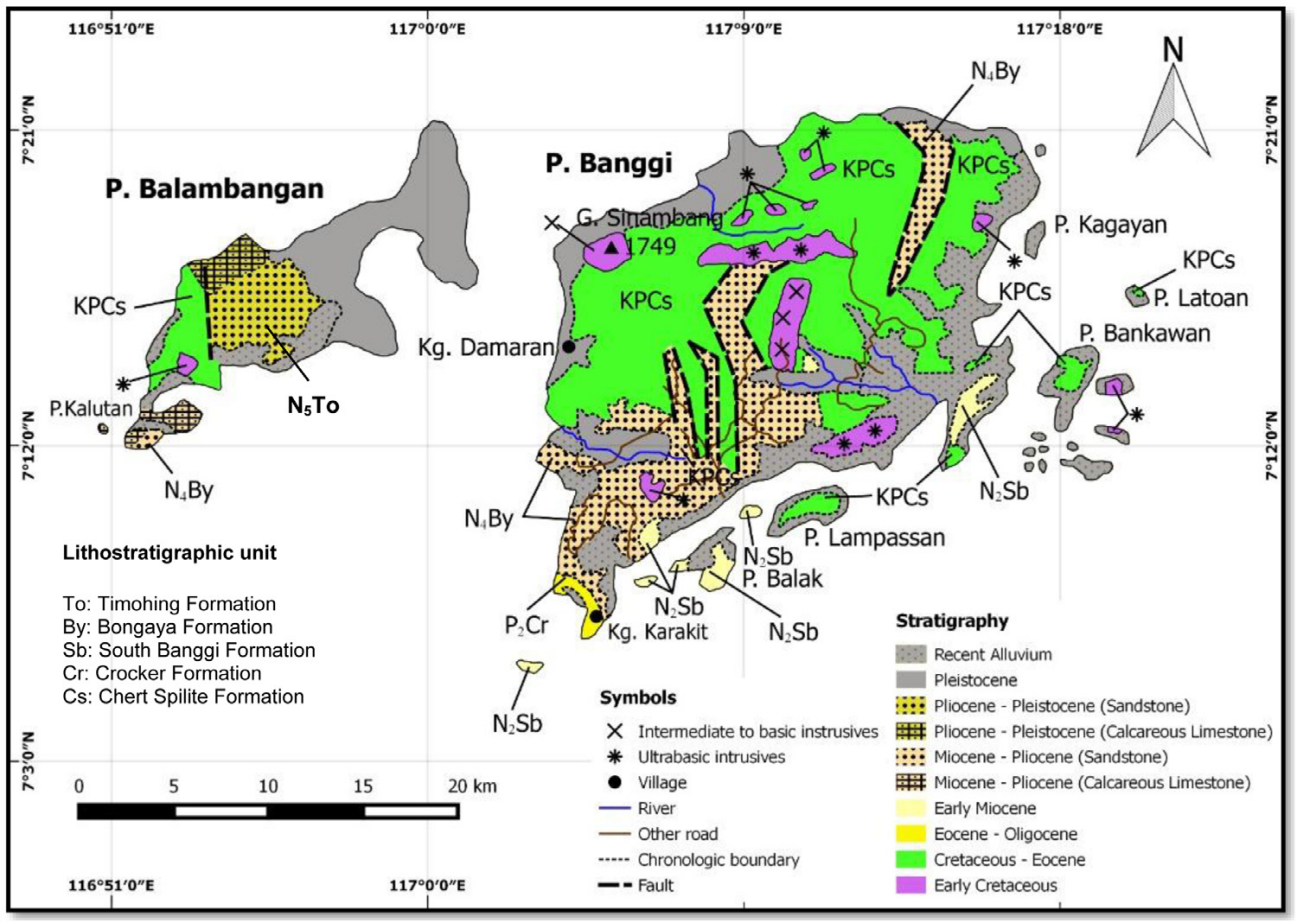
Geological map of Banggi Island. Slightly modified from the Department of Mineral and Geoscience Malaysia (JMG).

Banggi Island is located in Kudat, Sabah and is well-known as the largest island in Malaysia. It is located between longitude 117⁰04‘E to 115⁰17′E and latitude 7⁰06′N to 7⁰22′N (Fig. 1). The lithostratigraphic units bounded in the present study areas were generally suggested by [1, 2] to be represented as Bongaya and Chert-Spilite Formations (Fig. 1). Two areas were marked on both formations namely LPS and PPS. The LPS area was covered by the Bongaya Formation while PPS area was bounded by Chert-Spilite Formation.

### 3.2 Data acquisition

**Fig. 2:**
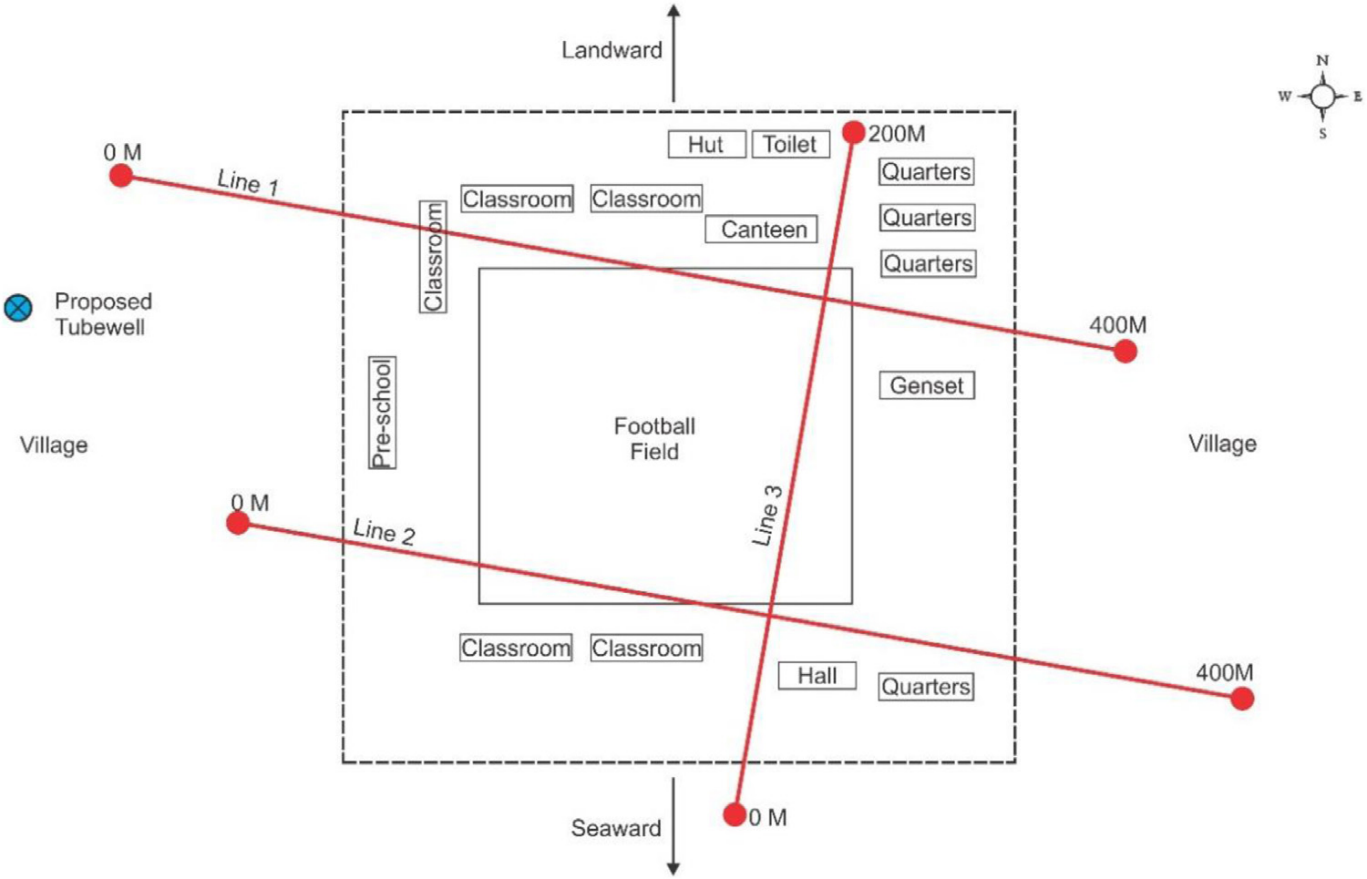
Layout plan of pole-dipole long short array survey lines within LPS area.

**Fig. 3:**
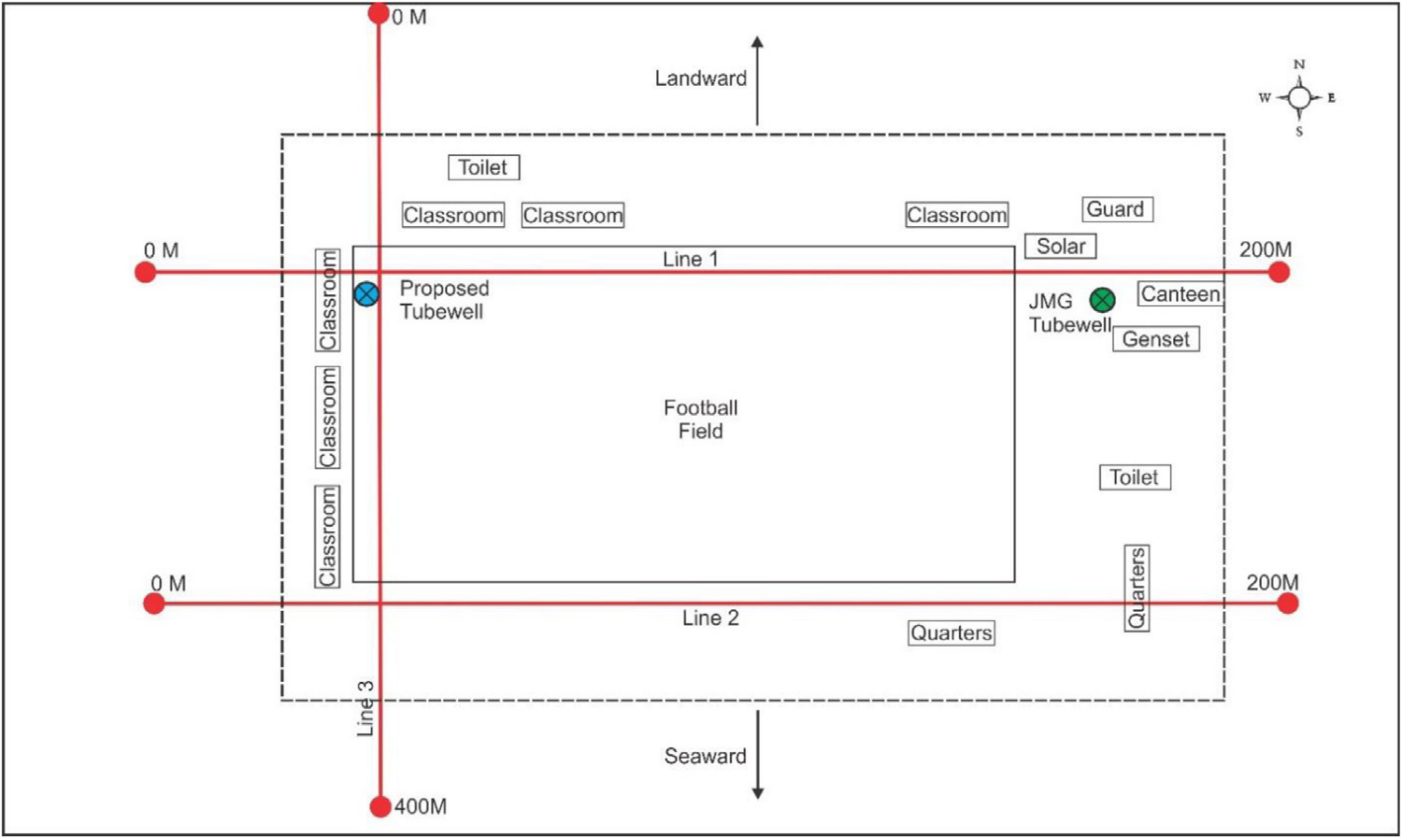
Layout plan of pole-dipole long short array survey lines within PPS area.

**Photo 1:**
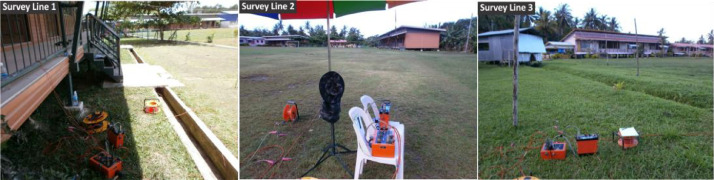
Terrameter SAS4000 in progress acquiring data from LPS area.

**Photo 2:**
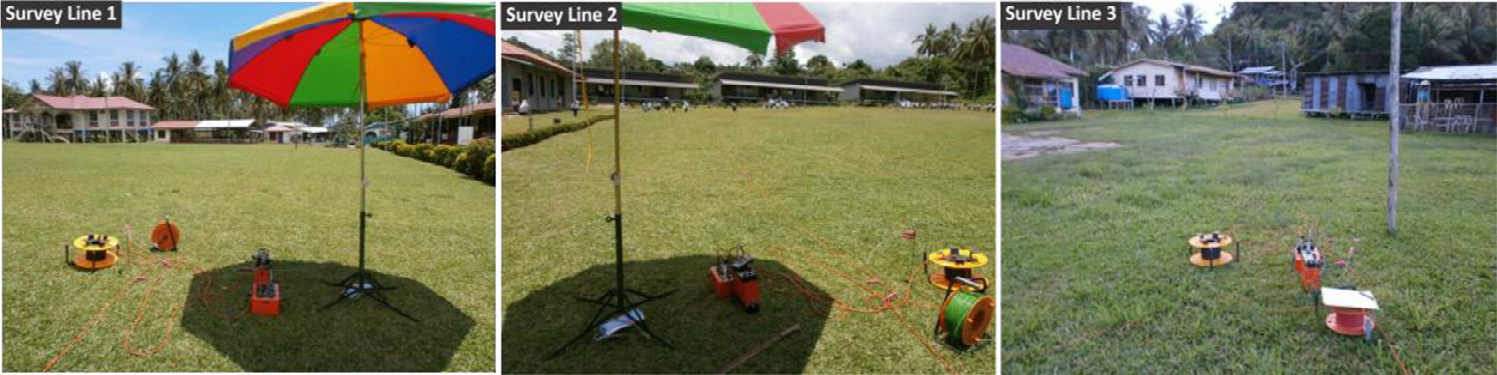
Terrameter SAS4000 in progress acquiring data from PPS area.

Fig. 2, Fig. 3 show the layout plan for 2D survey acquisition data of LPS and PPS. The complete resistivity tools used in the survey were SAS 4000 ABEM Lund Imaging System, together with a relay switching unit (Electrode Selector ES 464), one 220-m, two 200-m and three 400-m multiconductor cables, steel rod electrodes and jumpers. A pole-dipole long short array was also used for the survey (Photos 1 and 2). The pole-dipole array had relatively good horizontal coverage, but it had a significantly higher signal strength compared with the dipole-dipole array, though it was not as sensitive to telluric noise as a pole-pole array. Every transect line employed different electrode, cable jumper and lund cables, i.e. for every 400 metres, the stretch utilised 61 electrodes, 64 cable jumpers and 4 roll lund king cables. Each 200 to 220-metres stretch utilised 41 electrodes with 42 cable jumpers and 2 roll lund imaging cables. The field procedure involved certain parameters like electrode spacing, depth of investigation, subsurface resistivity, type of material and horizontal data coverage [3].

**Fig. 4:**
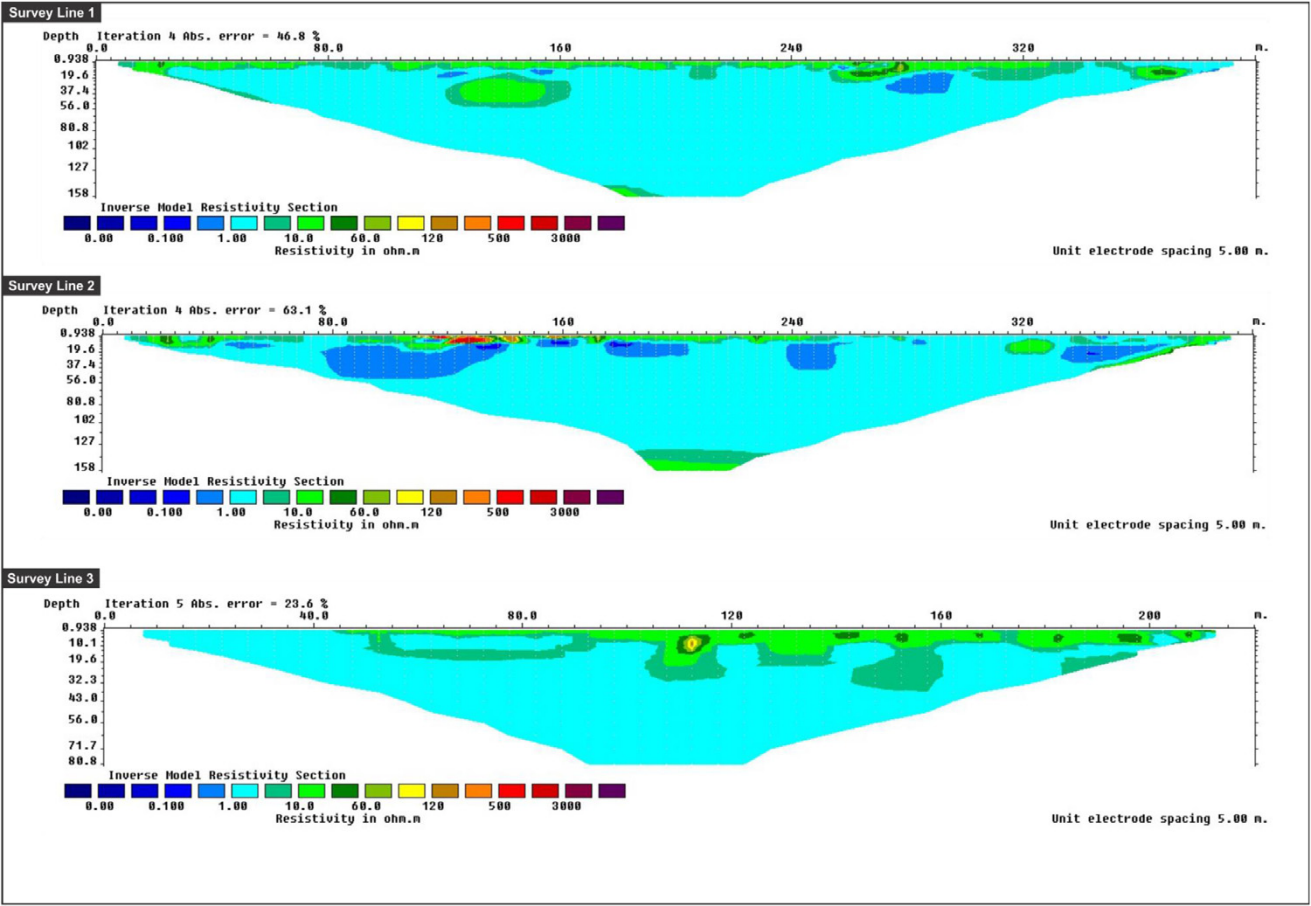
An Inverse model resistivity sections along survey line 1 to 3 of LPS area.

### 3.3 Data processing

Apparent resistivity data were processed by using RES2DINV (ver. 3.71.11). The software used smoothness-constrained Gauss-Newton least-squares inversion technique [4]. A 2D resistivity model was produced automatically by dividing the subsurface into rectangular blocks and selecting the optimum inversion parameters for the data including the damping factor, vertical to horizontal flatness filter ratio, convergence limit, and some iterations [5]. In addition, the inversion parameters were adjusted to suit the different data types. The software calculated the apparent resistivity values of the model blocks using either a finite difference or finite element method and compared them to measured data. The resistivity of the model blocks was modified iteratively until the measured perceived resistivity values of the model were in accordance with the real measurement values [6]. The software produced both a pseudo-section, which was a numerical means of describing the spatial variations of the measured or computed apparent resistivities, and an inverse model section, which was a tomogram displaying the modelled depth and shape resistivities.

**Fig. 5:**
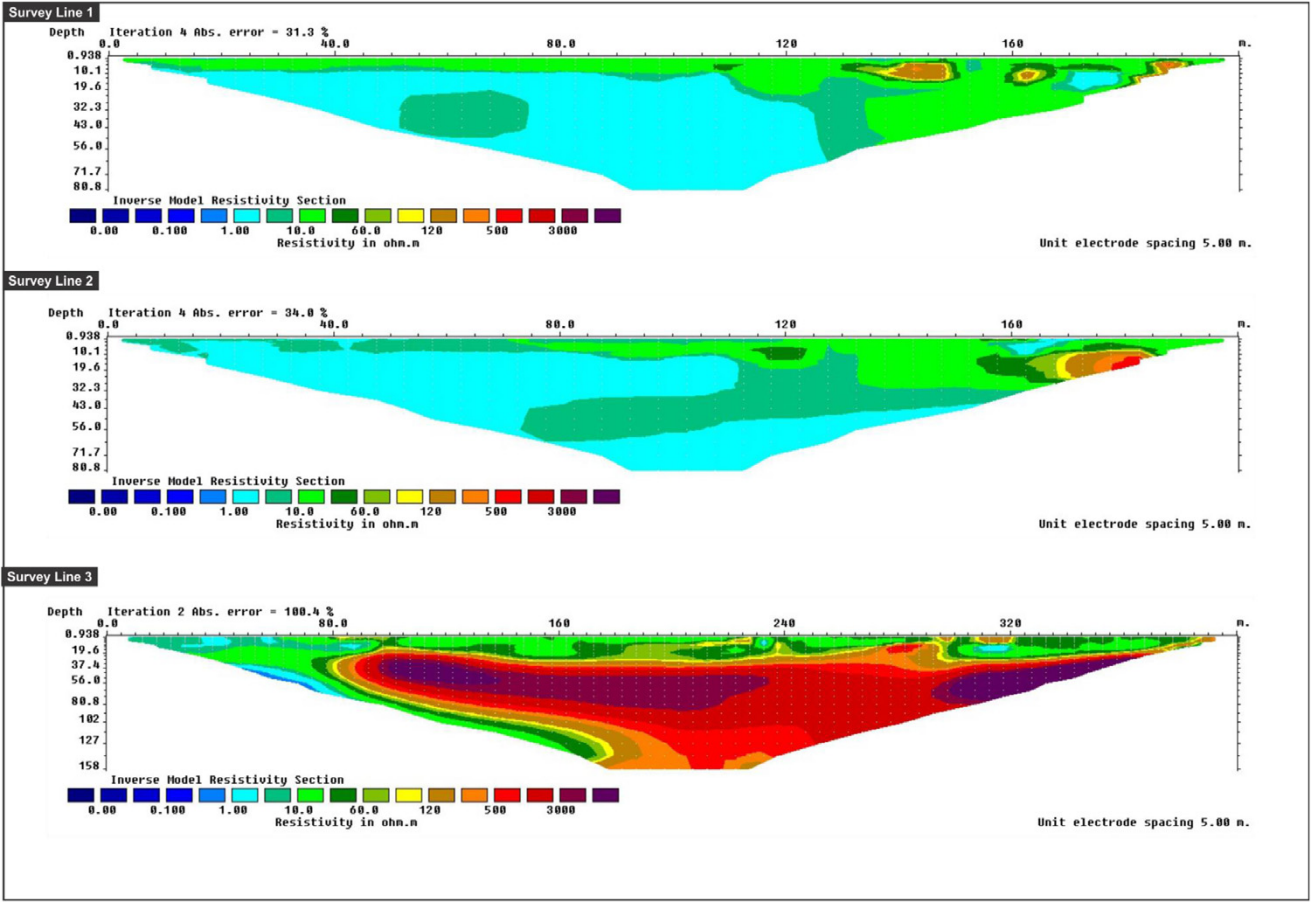
An Inverse model resistivity sections along survey line 1 to 3 of PPS area.

Fig. 4 shows Lines 1, 2 and 3 of LPS's inverse model resistivity section. Lines 1 and 2 were established as horizontal E-W direction with 400 metres stretched laterally. The present survey produced a few horizons, namely surface water, sea water, clay, silt and freshwater zones. The surface water zone was exposed uniformly on the upper part of the pseudo-section, while the clay and silt zone marked the lower part of the pseudo-section as it was essential to build up the major subsurface soils. Sea water zone existed on a 280-metre stretch along the survey line indicating an intrusion caused by sea water. The aquifer could be seen at a depth of 150 metres, though only visible on a small-scale. The freshwater zone could be seen at a depth of 150 metres away from sea level. For Line 3, an inverse model resistivity section was established as a vertical N-S direction in LPS area where the transect line was stretched up to 220 metres vertically. Surface water zone can be observed in the upper part of the section, while the lower section was surrounded by a brackish water zone, which is formed due to the accumulation of sandy clay.

Fig. 5 shows an inverse model resistivity section as well as the pseudo-sections for the PPS area. Pseudo-section for survey Lines 1 and 2 revealed that the two zones, namely, surface water zone and impermeable zone, exposed accumulations from the 200-metre stretch in the E-W direction. However, survey Line 3 was aligned perpendicular to survey Lines 1 and 2 with a stretch of up to 400 metres in N-S vertical direction. This N-S transect showed high resistivity between depths of 25 to 150 metres. The pseudo-section displayed hard formation zone that consisted of thick bedded sandstone.

## Author contributions

**Muhd Nur Ismail Abdul Rahman** performed Conceptualization, Methodology, Formal analysis, Writing-original draft, visualization.

**Hafeez Jeofry** performed Writing-review and Editing.

**Mohd Sazaly Basarian** performed Software, investigation, Resources, Data curation

## Declaration of Competing Interest

The authors declare that they have no known competing financial interests or personal relationships which have, or could be perceived to have, influenced the work reported in this article.

## Appendix Supplementary materials

Image, application 1

Image, application 2

Image, application 3
